# A Purified Serine Protease from *Nereis virens* and Its Impaction of Apoptosis on Human Lung Cancer Cells

**DOI:** 10.3390/molecules22071123

**Published:** 2017-07-07

**Authors:** Yunping Tang, Fangmiao Yu, Guomei Zhang, Zuisu Yang, Fangfang Huang, Guofang Ding

**Affiliations:** 1 School of Food and Pharmacy, Zhejiang Provincial Engineering Technology Research Center of Marine Biomedical Products, Zhejiang Ocean University, 1st Haidanan Road, Changzhi Island, Lincheng, Zhoushan 316022, China; tangyunping1985@163.com (Y.T.); fmyu@zjou.edu.cn (F.Y.); 13516829796@163.com (G.Z.); abc1967@126.com (Z.Y.); gracegang@126.com (F.H.); 2 Zhejiang Marine Fisheries Research Institution, Zhoushan 316021, China

**Keywords:** *Nereis virens*, serine protease, lung cancer, apoptosis, mitochondrial dysfunction

## Abstract

Nereis active protease (NAP) is a novel fibrinolytic active serine protease from the polychaete *Nereis virens*. In this study, NAP was purified from *Nereis virens* and the effects of NAP on human lung cancer cells were investigated. Our results indicated that NAP inhibited the proliferation and induced apoptosis of H1299 cells in a time- and dose-dependent manner. The loss of mitochondrial membrane potential, the activation of Bax and cleaved-caspase 3/9, the release of cytochrome C, and the suppression of Bcl-2 and poly-ADP ribose polymerase were observed in NAP-treated H1299 cells by flow cytometry and Western blotting. Moreover, the expression levels of Bax and Bcl-2 mRNA were determined by real-time quantitative polymerase chain reaction and the Bax/Bcl-2 expression ratio was increased in the NAP-treated cell lines. The results indicated that NAP-induced apoptosis may be related to mitochondria mediated apoptosis and occurs through caspase-dependent pathways. Then, the effects of NAP on tumor growth in animal models were observed, where 5 or 10 mg/kg of NAP noticeably reduced tumor volume and weight and increased apoptosis as determined by Western blotting when compared to the negative control group. Therefore, our findings suggest that NAP could be a hopeful anticancer medicine for its propensity to inhibit growth and induce of apoptosis in human lung cancer cells.

## 1. Introduction

Lung cancer is one of the most common and fatal cancers in the world [1,2]. Approximately 1.61 million new cases are diagnosed each year, with 1.38 million deaths recorded worldwide [3,4].

Recent studies show that almost one third of all cancer-related death in males and one fourth of all cancer death in females are due to the cause of lung cancer. About 80% of lung cancers are classified as non-small cell lung cancer (NSCLC) and 20% are small cell lung cancer (SCLC). Recently, the incidence and mortality of lung cancer in China has also increased because of smoking and increased air pollution [5,6]. Currently, surgery, radiation, chemotherapy and targeted therapy are used in lung cancer treatment [7,8,9,10]. However, resistance to anticancer drugs has been observed and normal cells may also be destroyed by the side effects of anticancer drugs [11,12]. Therefore, it is necessary to study and develop more effective and low-toxicity natural anticancer drugs.

Apoptosis is one of the main mechanisms of cell death in tumor treatment [13,14]. Its deregulation, resulting from a loss of pro-apoptotic signals or an increase in anti-apoptotic signals, may lead to a variety of pathological conditions, such as the initiation, promotion, and progression of cancer, or lead to treatment failures [15]. Apoptosis has become a preferable choice of cancer cell death for the treatment of cancers because of it does not trigger inflammatory or immune responses. Recently, several anticancer or anti-proliferative peptides have been purified from marine-derived protein hydrolysates to induce apoptosis. For example, Gln-Pro-Lys, a tripeptide from *Sepia* ink protein hydrolyates, was found to inhibit the proliferation of prostate cancer cells in a time- and dose-dependent manner [16]. Phe-Ile-Met-Gly-Pro-Tyr, a hexapeptide from protein hydrolysates of skate (*Raja porosa*) cartilage, was found to induce apoptosis by upregulating the Bax/Bcl-2 ratio and activating caspase-3 in HeLa cells [7]. Ala-Val-Leu-Val-Asp-Lys-Gln-Cys-Pro-Asp, a decapeptide purified from *Ruditapes philippinarum* protein hydrolysates, was found to have inhibitory effects on breast, prostate, and lung cancer cell proliferation [13]. However, there were no references in relation to anti-lung cancer protease extracted from marine sources. In this study, purified *Nereis* serine protease (NAP) was obtained from *Nereis virens*, according to the previous study [17], and in vitro experiments carried out using lung cancer cells to determine the effectiveness of NAP in inducing apoptosis. Furthermore, in vivo experiments were also performed to determine whether NAP suppresses lung tumor growth. Our results indicated that NAP inhibited the proliferation of human lung cancer cells, especially H1299 cells, induced apoptosis and regulated protein expression related to apoptosis through mitochondrial dysfunction.

## 2. Results

### 2.1. Purification of NAP

**Figure 1:**
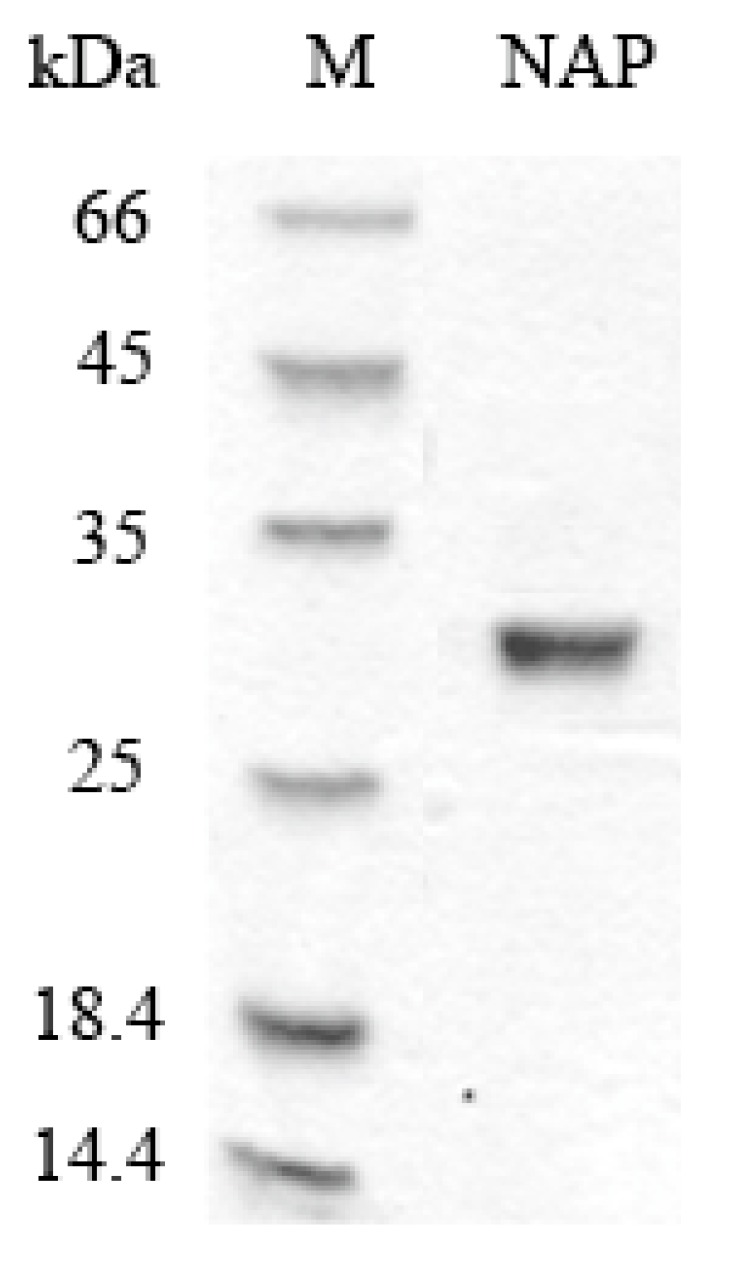
SDS-PAGE analyses of purified NAP. M: protein marker; NAP: purified NAP.

NAP was purified from *Nereis virens* through ammonium sulfate precipitation, anion exchange chromatography, and gel chromatography. Protease activity was used to monitor the purification. The results of the sodium dodecyl sulfate polyacrylamide gel electrophoresis (SDS-PAGE) analysis (Figure 1) showed that purified NAP was successfully obtained and its molecular weight is estimated to be about 29 kDa, which is consistent with the previous study [18]. The total recovery of NAP from *Nereis virens* was approximately 35.6%.

### 2.2. Anti-Proliferative Activity to Different Human Lung Cancer Cells

**Figure 2:**
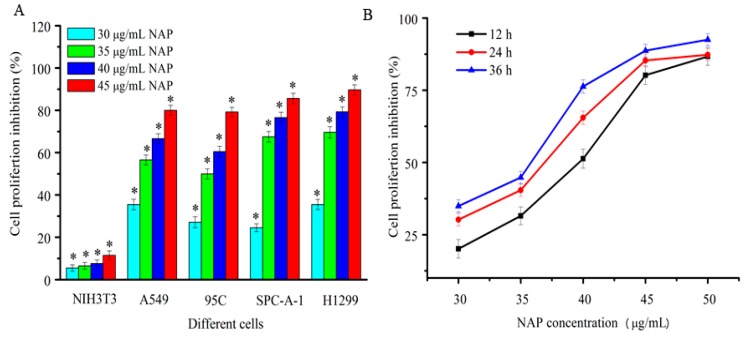
Inhibition of proliferation human lung cancer cells treated with NAP. (**A**) Proliferation inhibition of four human lung cancer cells treated by different NAP concentrations for 24 h; (**B**) Proliferation inhibition of H1299 cell lines treated with different NAP concentrations for 12, 24 and 36 h. * *p* < 0.05 vs. control.

In this study, four human lung cancer cell lines, A549, 95C, SPC-A-1, and H1299, were used to detect the proliferation inhibition of purified NAP by the MTT method. As shown in Figure 2A, NAP showed strong and dose-dependent cytotoxicity against human lung cancer cells after 24 h. The inhibition rate of A549, 95C, SPC-A-1, and H1299 cells was 80%, 79.2%, 85.6% and 89.7%, respectively, when treated with 45 μg/mL NAP after 24 h. NAP has almost no cytotoxic effects on normal cells because of the proliferation inhibition rate of it in NIH3T3 cells was far below than that in human lung cancer cells. Hence, the human non-small lung carcinoma H1299 cells were selected for further study. As shown in Figure 2B, NAP showed strong dose and time-dependent cytotoxicity against H1299 cells, with a half-maximal inhibitory concentration (IC50) of 40.1, 37.5 and 34.8 μg/mL at 12, 24, and 36 h, respectively.

### 2.3. Morphological Observations

**Figure 3:**
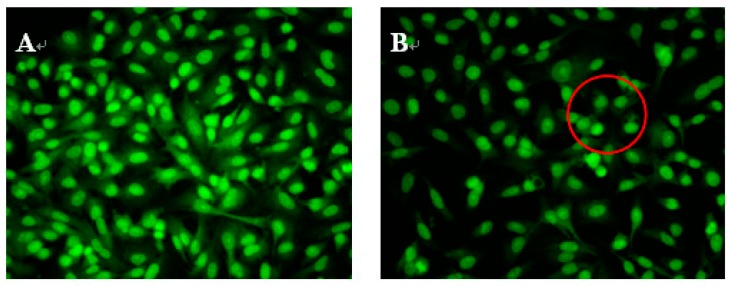

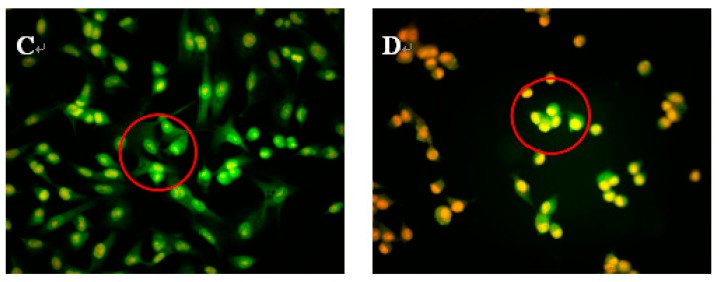
Morphological observation by AO/EB staining (200×). H1299 cells (**A**) were untreated, treated with 30 μg/mL NAP (**B**); with 40 μg/mL NAP (**C**); and with 50 μg/mL NAP (**D**). The red circle in Figure 3B indicates viable cells; the red circle in Figure 3C indicates early apoptotic cells; the red circle in Figure 3D indicates the late apoptotic cells. Each experiment has been done in triplicate (*n* = 3) and had similar morphological characteristics.

To study whether NAP’s inhibition of H1299 cell proliferation was attributable to apoptosis, H1299 cells were treated with 30, 40 or 50 μg/mL NAP, and the morphological changes of H1299 cells observed by acridine orange and ethidium bromide (AO/EB) staining and fluorescence microscopy (Figure 3). Green, yellow/green, and reddish/orange staining represented viable, early apoptotic and late apoptotic cells, respectively. As shown in Figure 3B,C, the yellow/green staining of H1299 cells was observed when treated with 30 and 40 μg/mL NAP after 24 h and indicated that the H1299 cells were in an early stage of apoptosis. Chromatin condensation, membrane blebbing, and fragmented nuclei were also discovered in H1299 cells after treatment with 30 and 40 μg/mL NAP for 24 h. In Figure 3D, additional features of apoptotic bodies of the orange necrotic cells were found, indicating that H1299 cells were at the final stages of apoptosis following treatment with 50 μg/mL of NAP for 24 h.

### 2.4. Cell Apoptotic Rate Detected by Flow Cytometry

**Figure 4:**
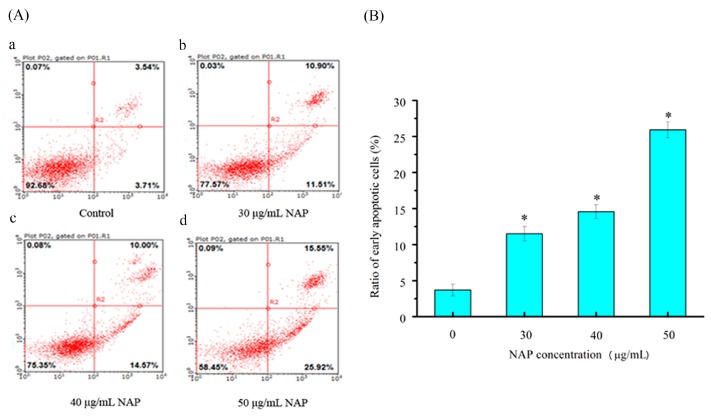
The apoptotic effect of NAP on H1299 cells. (**A**) Flow cytometry analysis of H1299 cells by double-labeling with Annexin-V fluorescein isothiocyanate (FITC) and PI. Annexin-V and propidium iodide (PI). Quadrants: lower left-live cells; upper left-necrotic cells; lower right-early apoptotic cells; upper right-late apoptotic cells. The percentage of early apoptotic cells was 3.71% (a) in the negative control cells; 11.51% (b) in the 30 μg/mL NAP-treated cells; 14.57% (c) in the 40 μg/mL NAP-treated cells and 25.92% (d) in the 50 μg/mL NAP-treated cells; (**B**) The percentage of apoptotic cells. All data are presented as the mean ± standard deviation (SD) of three experiments. * *p* < 0.05 vs. control.

To quantitatively measure the NAP-induced apoptotic process in H1299 cells, Annexin-V fluorescein isothiocyanate (FITC) and propidium iodide (PI) double staining was used to count the number of apoptotic cells [16]. As shown in Figure 4, the percentage of Annexin V-FITC-stained H1299 cells was 3.71 ± 1.4% for the control. After 24 h exposure to 30, 40, and 50 μg/mL NAP, the percentage of apoptotic cells increased to 11.51 ± 1.6%, 14.57 ± 1.8%, and 25.92 ± 2.1%, respectively. Obviously, the apoptotic effect on the H1299 cells was significantly increased with increasing NAP concentration, when compared to the control group. Therefore, NAP has a significant ability to induce apoptosis in H1299 cells.

### 2.5. Mitochondrial Membrane Potential (ΔΨm) Change in H1299 Cells Treated with NAP

**Figure 5:**
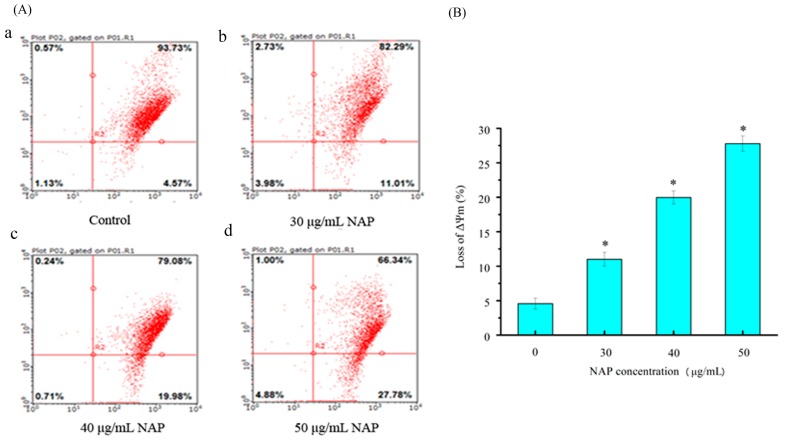
The effect of NAP on ΔΨm on H1299 cells. (**A**) Flow cytometry analysis of H1299 cells by the ΔΨm method. The percentage of ΔΨm change was 4.57% (a) in the negative control cells; 11.01% (b) in the 30 μg/mL NAP-treated cells; 19.98% (c) in the 40 μg/mL NAP-treated cells; and 27.78% (d) in the 50 μg/mL NAP-treated cells; (**B**) The percentage of loss of ΔΨm. All data are presented as the mean ± standard deviation (SD) of three experiments. * *p* < 0.05 vs. control.

External and internal pressures may cause a decrease in (ΔΨm) of cells, which can also induce apoptosis [18]. Changes in the (ΔΨm) were observed through JC-1 staining in a previous study [16]. In this study, H1299 cells stained with JC-1 were measured by flow cytometry. As shown in Figure 5, the percentage of JC-1 stained H1299 cells was 4.57 ± 1.2% for the control. After 24 h exposure to 30, 40 and 50 μg/mL NAP, the change of mitochondrial membrane potential was 11.01 ± 1.4%, 19.98 ± 2.1%, and 27.78 ± 2.3%, respectively. Our results demonstrated that NAP can reduce the mitochondrial membrane potential in H1299 cells and potentially lead to mitochondrial dysfunction.

### 2.6. Effects of NAP on the Apoptosis-Related Proteins of H1299 Cells

**Figure 6:**
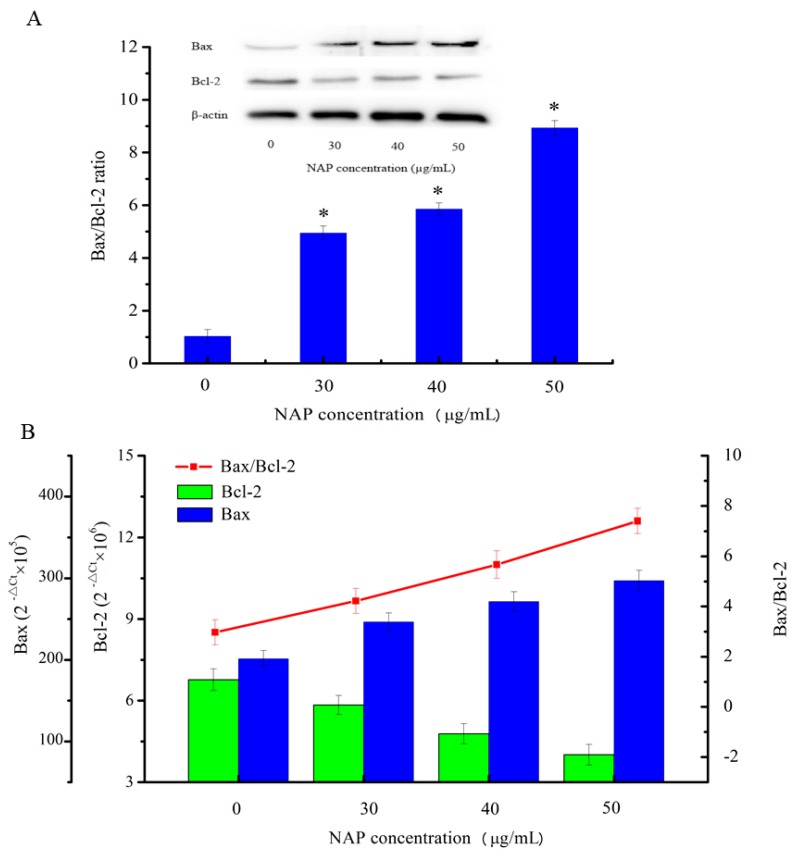
Expression of the apoptosis-associated proteins Bax and Bcl-2 in H1299 cells treated with NAP for 24 h. (**A**) Western blotting of Bax and Bcl2 expressed in H1299 cells treated with 0, 30, 40, and 50 μg/mL NAP and the Bax/Bcl2 ratio. The blots were detected with β-actin antibody to determine equal sample loading. * *p* < 0.05 vs. control; (**B**) The mRNA expression level of Bax and Bcl2 in H1299 cells treated with 0, 30, 40 and 50 μg/mL NAP and the Bax/Bcl2 ratio.

The results of the flow cytometry experiment showed that the apoptosis rate increased and the mitochondrial membrane potential reduced in H1299 cells with increasing NAP concentration. To understand the underlying mechanisms, western blotting was used to detect the apoptosis-related proteins involved in the mitochondrial dysfunction in H1299 cells in the present study. As shown in Figure 6A, the results indicated that with increasing NAP concentration, Bcl-2 expression was decreased and Bax expression was increased, eventually leading to an increase in the Bax/Bcl-2 ratio in NAP-treated H1299 cells. In parallel, as shown in Figure 6B, treatment of H1299 cells with 50 μg/mL of NAP for 24 h increased the Bax/Bcl-2 ratio at the mRNA expression level. The results indicated that NAP can promote apoptosis of H1299 cells by the upregulation of the Bax/Bcl-2 ratio.

**Figure 7:**
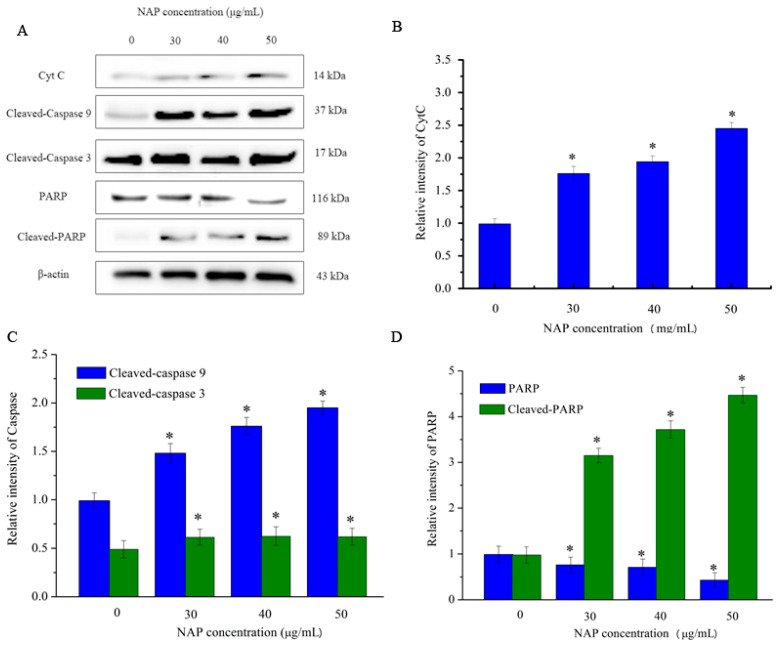
Expression of other apoptosis-associated proteins in H1299 cells treated with NAP for 24 h. H1299 cells were treated with 0, 30, 40 and 50 μg/mL NAP for 24 h and cells were harvested to measure (**A**) Western blotting of CytC, cleaved-caspase 9, cleaved-caspase 3, PARP, and cleaved-PARP protein; (**B**) Protein expression level of CytC that expressed in the treated-H1299 cells; (**C**) Protein expression level of cleaved-caspase 9 and cleaved-caspase 3 that expressed in the treated-H1299 cells (**D**) Protein expression level of PARP and cleaved-PARP that expressed in the treated-H1299 cells. The blots were also probed with β-actin antibodies to confirm equal sample loading. The blots were detected with β-actin antibody to determine equal sample loading. * *p* < 0.05 vs. control.

However, CytC and apoptosis-inducing factor are released into the cytoplasm when mitochondrial damage occurs. CytC may activate downstream caspases and lead to apoptosis [18]. As indicated in Figure 7, the expression level of CytC in H1299 cells increased with increasing NAP concentration. In addition, the expression level of cleaved-caspase 3/9 also increased. By contrast, the expression level of poly (ADP-ribose) polymerase (PARP) decreased and the expression level of cleaved-PARP increased. As cleaved-PARP can induce DNA breaks and lead to apoptosis, our results indicated that NAP-induced apoptosis is mediated by mitochondrial dysfunction.

### 2.7. Effects of NAP on Tumor Growth in an in Animal Models

**Figure 8:**
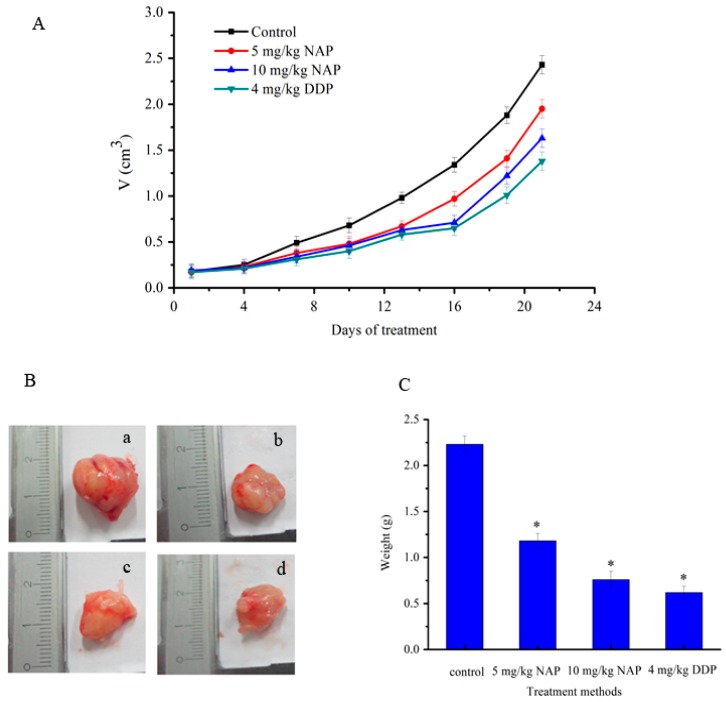
Inhibition of H1299 lung tumor growth by the NAP. (**A**) To identify the effect of NAP on H1299 lung tumor growth, nude mice were treated with 0, 5 and 10 mg/kg NAP for 21 days (*n* = 4). The positive control was treated with 4 mg/kg DDP; (**B**) Pictures of the final tumors. (a) Nude mice treated with 0 mg/kg NAP. (b) Nude mice treated with 5 mg/kg NAP. (c) Nude mice treated with 10 mg/kg NAP. (d) Nude mice treated with 4 mg/kg DDP; (**C**) The graph shows the final weight of the tumor. * *p* < 0.05 vs. control.

The effects of NAP on tumors caused by transplanting H1299 cells into nude mice were also evaluated in this study. Tumor size was determined every three days and NAP diluted with PBS and administered at 5 or 10 mg/kg body weight every three days by intraperitoneal injection. Tumor growth was relatively slow in the drug group, and was slower in the positive drug group when compared with the control (Figure 8A). At twenty-one days, tumors in the low concentration group (5 mg/kg of NAP) were 19.8% smaller, while those in the high concentration group (10 mg/kg of NAP) were 32.9% smaller when compared to the control (Figure 8A). The tumors in the positive drug group (4 mg/kg of cisplatin (DDP)) were 43.2% smaller than the tumors in the negative control group (Figure 8A). However, the average weight of tumors in mice that received 5 mg/kg of NAP was 1.18 ± 0.13 g, 0.76 ± 0.09 g in those receiving 10 mg/kg of NAP and 0.62 ± 0.07 g in mice receiving 4 mg/kg of DDP. However, there was a clear trend toward decreasing tumor weight when compared to the negative control, in which tumors weighed 2.23 ± 0.16 g on average (Figure 8B,C).

### 2.8. Effects of NAP on the Apoptosis-Related Proteins of H1299 Tumor Tissue

**Figure 9:**
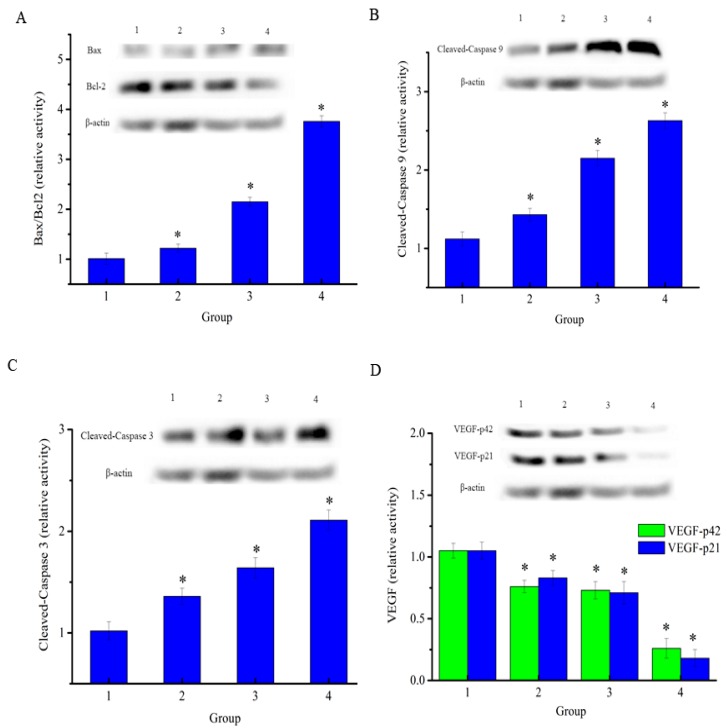
Expression of apoptosis-associated proteins in H1299 cells treated with NAP or DDP. (**A**) Western blotting of Bax and Bcl2 expressed in the treated-H1299 cells and the Bax/Bcl2 ratio; (**B**) Western blotting of cleaved-caspase 9 that expressed in the treated-H1299 cells; (**C**) Western blotting of cleaved-caspase 3 that expressed in the treated-H1299 cells; (**D**) Western blotting of VEGF that expressed in the treated-H1299 cells. The blots were detected with β-actin antibody to determine equal sample loading. * *p* < 0.05 vs. control. 1: without NAP; 2: treated with 5 mg/kg NAP; 3: treated with 10 mg/kg NAP; 4: treated with 4 mg/kg DDP.

To determine the anticancer effects on H1299 lung cancer, the drug was injected in mice carrying xenograft tumors. At the end of the experiment, mice were euthanized and the tumors removed and cracked by liquid nitrogen grinding to extract the total proteins. As shown in Figure 9A, the results showed that the expression level of Bcl-2 was decreased and the expression level of Bax was increased in both the NAP-treated groups and positive control drug group when compared to the negative control. The Bax/Bcl-2 ratio in NAP-treated and DPP-treated H1299 tumors increased when compared to the negative control. In parallel, as shown in Figure 9B, NAP clearly upregulated the expression level of cleaved-caspase 9 in tumors and its relative intensity increased from 1.12 to 2.15 when the NAP concentration ranged from 0–10 mg/kg. Figure 9C shows that NAP also clearly upregulated cleaved-caspase 3 levels in tumors and its intensity increased from 1.02 to 1.63 over a NAP concentration range of 0–10 mg/kg. NAP significantly inhibited the expression of vascular endothelial growth factor (VEGF) levels in tumors when compared with the control group (Figure 9D).

## 3. Discussion

Lung cancer is one of the most common malignancies that seriously threatens human life [19]. Currently, drugs used in cancer treatment are not only toxic to cancer cells, but also toxic to normal cells [20]. However, natural products exist which have low toxicity and may represent good candidates for inhibiting tumor cell growth [21]. In this study, an anti-lung cancer protein NAP was purified from *Nereis virens*. NAP showed strong, dose-dependent cytotoxicity against human lung cancer cells (A549, 95C, SPC-A-1, and H1299) after 24 h. Further investigation revealed that NAP showed strong, dose- and time-dependent cytotoxicity against H1299 cells.

Apoptosis, or programmed cell death, is a key regulator of physiological growth regulation and tissue homeostasis [22]. Apoptosis has typical morphological and biochemical characteristics, including fragmentation of nuclear DNA, cell shrinkage, and cell membrane blebbing [23]. These changes can be observed using fluorescence microscopy and AO/EB staining, and are usually used to distinguish between apoptotic and normal cells [24,25]. The effects of NAP on cell apoptosis were evaluated by AO/EB staining and the results revealed that the morphologic features of apoptotic H1299 cells were dependent on the dose of NAP, as observed in previous studies [26]. Annexin-V FITC can bind to phosphatidylserine, which is distributed only on the inner side of the cell membrane and transfers to the outer side of the cell membrane during early cell apoptosis. Thus, Annexin V-FITC was used to indicate cells entering apoptosis and PI was used to determine the number of necrotic cells. Flow cytometry revealed that the percentage of apoptotic cells was significantly increased with increasing NAP concentration in a dose-dependent manner. The results in this study indicated that NAP exhibits anti-lung cancer activity through the induction of apoptosis.

The possible mechanism for apoptosis induced by NAP was investigated in this study. Mitochondrial damage has been shown to play an important role in cell apoptosis in the previous studies [27]. The loss of ΔΨm is an important parameter of mitochondrial damage. Recent studies have showed that some apoptotic stimuli lead to the release of calcium ions in the endoplasmic reticulum (ER) stores, causing mitochondrial calcium ions overload, inducing the mitochondrial permeability transition, and mitochondrial matrix swelling [28]. Therefore, the outer mitochondrial membrane begins to rupture and pro-apoptotic intermembrane space proteins escape into the cytoplasm. Our results demonstrated that human lung cancer cells treated with NAP had a significant loss of ΔΨm when compared with the control. The Bcl-2 family, which is comprised of proteins that change the permeability of the mitochondrial membrane, plays an important role in the regulation of apoptosis [29]. Bcl-2-family proteins also regulate the Ca^2+^ pathway through their localization at the ER. For example, Bcl-2, which acts as anti-apoptotic regulators, can reduce the releasable pool of ER Ca^2+^ and desensitize cells to death by C2-ceramide. Bax, which possess pro-apoptotic characteristics, acts in the opposite manner and increases the amount of Ca^2+^ releasable from the ER [28,30]. In this study, we showed that NAP treatment resulted in an increase of the Bax/Bcl-2 ratio and the Bax/Bcl-2 mRNA expression ratio was also increased in the NAP treated H1299 cells, which may cause Ca^2+^ overload of the mitochondria and decrease the mitochondrial membrane potential, indicating that Bcl-2 family proteins participate in the control of NAP-induced mitochondrial damage. Furthermore, the disruption of mitochondrial membrane potential could lead to the release of CytC and activation of caspases in mitochondria-dependent pathways. Our results showed that CytC protein levels increased with increasing NAP concentration, and the levels of cleaved-caspase 3/9 proteins were also increased. By contrast, the expression of PARP was decreased and the expression of cleaved-PARP was increased, and then the cleaved-PARP induces DNA breaks. The above results suggested that NAP induced apoptosis of human lung cancer H1299 cells by mitochondria-dependent pathways.

Furthermore, the effects of NAP on tumors caused by transplanting H1299 cells into nude mice were evaluated in this study. Tumor growth was relatively slow in the drug treated group, and slower in the positive control drug group compared to the negative control group. Western blotting indicated that the Bax/Bcl-2 ratio was increased in NAP-treated and DDP-treated H1299 tumors. NAP also noticeably upregulated cleaved-caspase 3 and cleaved-caspase 9 levels in tumor cells. In lung cancer, VEGF plays an important role in establishing a vascular supply within the tumor [31]. VEGF promotes vascular endothelial growth and mediates vascular permeability, thereby promoting tumor progression and metastatic diffusion [32]. Our results demonstrated that NAP significantly inhibited the VEGF expression in tumors compared with the control group. These findings suggested that NAP restricts cancer cell growth by suppressing blood vessel formation.

Studies by Chen Hong et al reported a fibrinolytic enzyme found in earthworms exhibits effective anti-tumor activity in hepatoma cells both in vitro and in vivo [33]. The mechanism of enzyme activity may be through the induction of apoptosis in hepatoma cells and the inhibition of the expression of matrix metalloproteinase. Workers from Jinlin University reported that acid serine protease from *Neanthes japonica* can strongly inhibit the proliferation and viability of acute promyelocytic leukemia NB4 cells and chronic myeloid leukemia K562 cells in vitro. The mechanism may be related to the direct cytotoxic effect and induction of apoptosis [34,35]. However, our results confirmed that NAP could inhibit the proliferation of lung cancer H1299 cells in a time- and dose-dependent manner and induced cells apoptosis, similar to previous results. The mechanism of NAP-induced apoptosis may be related to mitochondria-mediated apoptosis and occurs through caspase-dependent pathways. Our future research will focus on the molecular and proteomics mechanisms of cell apoptosis induced by NAP, cloning of the NAP gene and heterologous expression of the gene in recombinant *E. coli* or *P. pastoris*.

**Figure 10:**
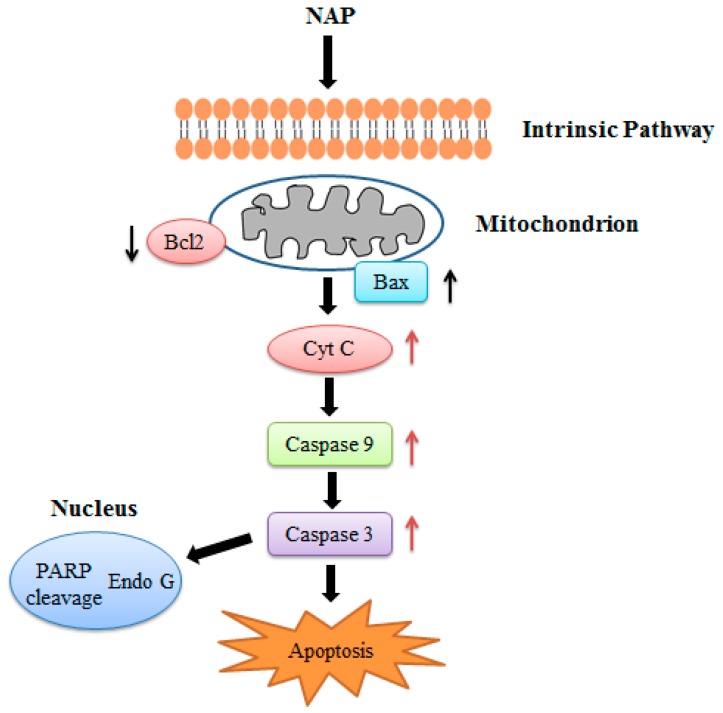
NAP induced apoptosis in the human lung cancer H1299 cells.

In conclusion, we confirmed the anticancer effects of NAP on human lung cancer H1299 cells both in vitro and in vivo. NAP suppressed the proliferation of H1299 cells, induced apoptosis, and regulated the protein expression related to apoptosis through mitochondrial dysfunction (Figure 10). Injection with 5 or 10 mg/kg NAP in mice carrying xenograft tumors could significantly reduce tumor volume and weight. Western blotting results also indicated that the Bax/Bcl2 ratio, cleaved-caspase 3/9 were increased and the expression levels of VEGF were decreased in tumors, indicating that apoptosis of H1299 lung cancer cells results from regulation of the intrinsic pathway. Thus, our findings show that NAP may be a potential natural drug for the treatment of human lung cancer.

## 4. Materials and Methods

### 4.1. Chemicals and Reagents

A methylthiazolyldiphenyl-tetrazolium bromide (MTT) cell proliferation and cytotoxicity assay kit was purchased from Beyotime Biotechnology (Shanghai, China). Annexin-V fluorescein isothiocyanate (Annexin-V FITC) and propidium iodide (PI) apoptosis detection kits were purchased from Sigma-Aldrich Trading Co., Ltd. (Shanghai, China). Antibodies against β-actin, Bax, Bcl-2, cytochrome C (CytC), caspase-9, caspase-3, and PARP were purchased from Beyotime Biotechnology (Shanghai, China). Unless specifically stated, all other reagents were analytical grade.

### 4.2. Cell Lines and Culture

Mouse embryo fibroblast NIH3T3 cells and human lung cancer cells (A549, 95C, SPC-A-1, and H1299) were purchased from the Cell Bank of Chinese Academy of Sciences. Cells were cultured in RPMI-1640 medium supplemented with 10% fetal bovine serum (FBS), 100 U/mL penicillin G sodium and 100 U/mL streptomycin sulfate. All cells were static incubated at 37 °C in a humidified 5% CO_2_ atmosphere incubator (Forma 3111, Thermo, Waltham, MA, USA) and the culture medium was changed every two to three days.

### 4.3. Purification of NAP

The purified NAP was extracted from *Nereis virens* according to the protocol of Zhang et al. with some modifications [17]. Firstly, samples were homogenized in 200 mL of Tris-HCl (0.1 M, 7.0) and placed for 30 min before use. hen, homogenate was centrifuged and supernatant was recovered and precipitated by the addition of 30% ammonium sulfate. The precipitate was recovered by centrifugation at 12,000 rpm for 10 min and re-dissolved in Tris-HCl (0.1 M, 7.0). The solution was then dialyzed at 4 °C overnight with several changes of Tris-HCl (0.1 M, 7.0) buffer and concentrated using PEG20000. The concentrated protein was then purified by anion exchange and gel chromatography with an AKTA purifier. The concentrated protein (5 mL) was added to a DEAE Sepharose Fast Flow column (2.0 cm × 15 cm), which had been pre-equilibrated with Tris-HCl (0.1 M, 7.0). The active penetrable fractions were collected and then concentrated by PEG 20,000. The concentrated protein (2 mL) was further added to a Sephadex G-25 column (2.6 cm × 90 cm) pre-equilibrated with Tris-HCl (0.1 M, 7.0) and eluted at a flow rate of 1 mL/min. All steps were performed at 4 °C and purified NAP was identified by SDS-PAGE. Protease activity was determined as described previously [17].

### 4.4. Anti-Proliferative Activity to Different Human Lung Cancer Cells

Anti-proliferative activity of normal cells and human lung cancer cells was determined using an in vitro MTT assay as outlined in previous study [16]. Cells were then seeded in a 96-well flat-bottomed plate (1 × 10^4^ cells/well) and allowed to adhere to the bottom of the wells for 24 h and treated with NAP at final concentrations of 0, 30, 35, 40, 45 or 50 μg/mL for another 24 h at 37 °C in a 5% CO_2_ incubator. Absorbance values were measured by using a microplate reader at 490 nm. The cell proliferation inhibition rate (%) was calculated using the follow equation: inhibition rate (%) = (1 − (A·treated/A control)) × 100%.

### 4.5. Cell Morphologic Study

H1299 cells were seeded in a six-well flat-bottomed plate (1 × 10^5^ cells/well) and cells were allowed to adhere to the bottom of the wells for 24 h prior to NAP treatment. Cells were treated to 0, 30, 40 and 50 μg/mL NAP for another 24 h, after which 25 μL of AO/EB dye mixture (100 μg/mL) in phosphate-buffered saline (PBS, pH 7.4) was added to the NAP-treated cells. Following staining, cells were imaged immediately under a fluorescence microscope (Leica DM 3000, Wetzlar, Germany).

### 4.6. Cell Apoptosis Analysis

Apoptosis rates were measured by flow cytometer using Annexin-V FITC/PI staining [16]. H1299 cells were firstly seeded in six-well flat-bottomed plates and incubated for 24 h. Cells were then treated with 30, 40 and 50 μg/mL NAP for another 24 h and harvested. After digestion with trypsin, the cells were suspended in PBS and harvested by centrifugation at 1000 rpm for 10 min. Then, cells were incubated with Annexin-V FITC (5 μL) and PI (10 μL) at room temperature for 15 min in the dark. After staining, cells were analyzed immediately by flow cytometry (Becton Dickinson, NJ, USA).

### 4.7. ΔΨm Change Effected by NAP

H1299 cells were seeded in six-well flat-bottomed plates and following 24 h incubation, cells were treated with 30, 40 and 50 μg/mL NAP for 24 h then harvested. Following digestion with trypsin, the cells were suspended in PBS and then harvested by centrifugation at 1000 rpm for 5 min. The ΔΨm change of treated H1299 cells was achieved using the protocol outlined in the mitochondrial membrane potential assay kit (JC-1) (Nanjing KeyGen Biotech. Co. Ltd., Nanjing, China). Cells were analyzed immediately by flow cytometry.

### 4.8. Western Blot Analysis

Total protein was extracted with RIPA lysis buffer and protein concentrations were determined using the BCA assay kit. Proteins were then separated by SDS-PAGE and transferred to a polyvinylidene difluoride membrane. After blocking with 5% skimmed milk for 1.5 h, the membrane was incubated with specific primary antibody and then with secondary antibody (horseradish peroxidase-conjugated goat-anti-rabbit, 1:3000) at room temperature for 2 h. The specific band intensity was detected by enhanced chemiluminescence method and quantified using Quantity One 4.62 software (Bio-Rad, Hercules, CA, USA) and compared to a known standard (β-actin).

### 4.9. RNA Isolation and Real-Time Quantitative Polymerase Chain Reaction (RT-PCR)

Total RNA and cDNA templates from H1299 cells or treated H1299 cells were prepared according to the previous study’s protocol [36]. Primers used to amplify gene targets are listed in Table 1. The total volume of amplification was 25 μL that containing 12.5 μL of PCR reaction mixture (2×), 1 μL (10 μM) of each target primer, 1 μL of template, and 9.5 μL of Diethy pyrocarbonate (DEPC) treated water. The PCR cycling program had an initial cycle of 94 °C for 3 min, followed by 30 cycles of 94 °C for 45 s, 55 °C for 30 s, 72 °C for 1 min, and a final step of 72 °C for 5 min. Ct values were obtained automatically using software (Bio-Rad, USA). The expression levels of Bax and Bcl-2 were analyzed using the comparative Ct method (2^−∆*C*t^ method). The primer pairs provided in the reagent box were used to amplify the housekeeping gene (glyceraldehyde-3-phosphate dehydrogenase).

### 4.10. In Vivo Antitumor Activity

Female BALB/c nude mice (nu/nu) were purchased from the Shanghai Laboratory Animal Center, Chinese Academy Sciences. H1299 cells were cultured in RPMI-1640 (10% FBS, 100 U/mL streptomycin sulfate and 100 U/mL penicillin G sodium) at 37 °C in a 5% CO_2_ incubator. H1299 cells were then harvested and treated with 0.25% trypsin. The cells were washed twice and resuspended in RPMI-1640, after which 0.15 mL of medium (with 1 × 10^7^ cells) were injected subcutaneously into the right flank of the donor nude mice. H1299 cells grew under the skin of nude mice and tumors were established seven days following injection. Formed tumors were measured and mice randomly divided into three treatment groups (two groups with 5 and 10 mg/kg NAP, a positive control group with 4 mg/kg DDP) and a negative control group in a blinder.

A digital caliper was used to measure the perpendicular diameters of tumors every three days and the tumor volume (mm^3^) was then calculated using the following formula: volume = π/6 × width × width × length. All animals were euthanized and tumors removed and weighed at the endpoint of the experiment. All experiments were performed in accordance with institutional animal care and use committee guidelines of Zhejiang Ocean University and adhered to The Code of Ethics of the World Medical Association (Declaration of Helsinki).

### 4.11. Statistical Analysis

All results are expressed as the mean ± standard deviation (*n* = 3). Experimental data were analyzed by a one-way analysis of variance (ANOVA) test using the SPSS 19.0 software. Significant differences were determined using Dunnett’s *t*-tests (*p* < 0.05 and 0.01).

## Figures and Tables

**Table 1:** Real-time PCR primers and conditions.

Gene	Genebank Accession	Primer Sequence	Product Length (bp)	Annealing (°C)
Bcl-2	NM-000657.2	F:5′-CCCGTTGCTTTTCCTCTGG-3′	1207	62.6
		R:5′-ATCCCACTCGTAGCCCCTCT-3′		
Bax	NM-138764.3	F:5′-GACGAACTGGACAGTAACATGGA-′3	849	61.4
		R:5′-GCAAAGTAAAAGGGCGACA-′3		
